# New psychometric analysis and normative data for the Argentinean Psycholinguistic Naming test in color: PAPDIC

**DOI:** 10.1002/alz.092474

**Published:** 2025-01-03

**Authors:** Leticia Vivas, Laura Manoiloff, Patricia Paolantonio, Miguel Horacio Lamenza, Carla Andrea Aragones, Maria Celeste López Moreno

**Affiliations:** ^1^ IPSIBAT (CONICET/National University of Mar del Plata), Mar del Plata, Buenos Aires Argentina; ^2^ Córdoba National University, Córdoba Argentina; ^3^ Universidad Internacional de La Rioja, Logroño Spain; ^4^ IIPSI (CONICET‐National University of Córdoba), Córdoba Argentina; ^5^ National University of Mar del Plata, Mar del Plata Argentina; ^6^ IPSIBAT (UNMDP/CONICET), Mar del Plata, Buenos Aires Argentina

## Abstract

**Background:**

Naming test are a very common tool in neuropsychological batteries. Some years ago, the Argentinean Psycholinguistic Naming test was developed, first with black and white drawings (Manoiloff et al., 2018) and then in colors (PAPDIC) (Vivas et al., 2020). Items were selected and organized on the basis of Argentinean psycholinguistic norms. At that time evidence of validity was reported (concurrent validity and contrasted groups analyses). Here we present new psychometric analysis and normative data for the Argentine population.

**Method:**

The sample comprised 292 participants: 195 healthy, 70 MCI, 14 stroke patients without aphasia and 13 stroke patients with aphasia. They went through a complete neuropsychological battery to ensure that no other cognitive function alteration (apart from language) could interfere with the task. Items were rated as dichotomous responses, and the final score only included those correct responses given before the phonological cue.

**Result:**

o assess the test’s internal consistency a KR20 analysis was performed (as items were dichotomous) which showed a value of .879. Besides, a one factor ANOVA was performed to compare the full test score between groups and significant differences were observed (F(3,288) = 51.867; p < .001). Particularly, healthy participants differentiate from the MCI (p < .001) and stroke with aphasia (p < .001) groups, but no with stroke patients without aphasia (p = .133). Another comparison was performed between this version of the test and a previous black and white version by a chi‐squared analysis with each item, and it showed a similar performance for 24 items, a better performance in the color version for 5 items and a worse performance in one item. Additionally, a regression analysis was performed in order to obtain normative data for the Argentine population. As age was the only demographic variable that showed significant effect on the test score (R2 adjusted = .140; F(1,192) = 32.545; p < .001), it was considered for this calculus.

**Conclusion:**

These results show that the PAPDIC is a reliable naming test for the Argentine population that can be used both for patients with neurodegenerative diseases and post stroke aphasia.

